# Dexamethasone and Methylprednisolone Promote Cell Proliferation, Capsule Enlargement, and *in vivo* Dissemination of *C. neoformans*

**DOI:** 10.3389/ffunb.2021.643537

**Published:** 2021-02-10

**Authors:** Glauber R. de S. Araújo, Vinicius Alves, Pedro H. Martins-de-Souza, Allan J. Guimarães, Leandro Honorato, Leonardo Nimrichter, Christina Maeda Takiya, Bruno Pontes, Susana Frases

**Affiliations:** ^1^Laboratório de Ultraestrutura Cellular Hertha Meyer, Instituto de Biofísica Carlos Chagas Filho, Universidade Federal Do Rio de Janeiro, Rio de Janeiro, Brazil; ^2^Laboratório de Biofísica de Fungos, Instituto de Biofísica Carlos Chagas Filho, Universidade Federal Do Rio de Janeiro, Rio de Janeiro, Brazil; ^3^Laboratório de Bioquímica e Imunologia das Micoses, Depto. de Microbiologia e Parasitologia, Instituto Biomédico, Universidade Federal Fluminense, Niterói, Brazil; ^4^Instituto de Microbiologia Paulo de Góes, Universidade Federal Do Rio de Janeiro, Rio de Janeiro, Brazil; ^5^Laboratório de Imunopatologia. Instituto de Biofísica Carlos Chagas Filho, Universidade Federal Do Rio de Janeiro, Rio de Janeiro, Brazil; ^6^Instituto de Ciências Biomédicas, Universidade Federal Do Rio de Janeiro, Rio de Janeiro, Brazil; ^7^Centro Nacional de Biologia Estrutural e Bioimagem (CENABIO), Universidade Federal Do Rio de Janeiro, Rio de Janeiro, Brazil

**Keywords:** *Cryptococcus neoformans*, glucocorticosteroids, methylprednisolone, dexamethasone, immune reconstitution inflammatory syndrome (IRIS), fungal virulence, capsule, polysaccharide

## Abstract

*Cryptococcus neoformans* is a fungal pathogen that causes life-threatening infections in immunocompromised individuals, who often have some inflammatory condition and, therefore, end up using glucocorticoids, such as dexamethasone and methylprednisolone. Although the effects of this class of molecules during cryptococcosis have been investigated, their consequences for the biology of *C. neoformans* is less explored. Here, we studied the effects of dexamethasone and methylprednisolone on the metabolism and on the induction of virulence factors in *C. neoformans*. Our results showed that both glucocorticoids increased fungal cell proliferation and surface electronegativity but reduced capsule and secreted polysaccharide sizes, as well as capsule compaction, by decreasing the density of polysaccharide fibers. We also tested whether glucocorticoids could affect the fungal virulence in *Galleria mellonella* and mice. Although the survival rate of *Galleria* larvae increased, those from mice showed a tendency to decrease, with infected animals dying earlier after glucocorticoid treatments. The pathogenesis of spread of cryptococcosis and the interleukin secretion pattern were also assessed for lungs and brains of infected mice. While increases in the spread of the fungus to lungs were observed after treatment with glucocorticoids, a significant difference in brain was observed only for methylprednisolone, although a trend toward increasing was also observed for dexamethasone. Moreover, increases in both pulmonary and cerebral IL-10 production, reduction of IL-6 production but no changes in IL-4, IL-17, and INF-γ were also observed after glucocorticoid treatments. Finally, histopathological analysis confirmed the increase in number of fungal cells in lung and brain tissues of mice previously subjected to dexamethasone or methylprednisolone treatments. Together, our results provide compelling evidence for the effects of dexamethasone and methylprednisolone on the biology of *C. neoformans* and may have important implications for future clinical treatments, calling attention to the risks of using these glucocorticoids against cryptococcosis or in immunocompromised individuals.

## Introduction

Fungal infections that cause systemic mycoses have become a major threat since the end of the 20th century. Although fungal infections are underreported or undetected due to limitations in the differential diagnosis (Perfect, 2013), the rise in number of fungal infections is closely associated with a significant increase in immunocompromised patients due to glucocorticoid therapy, immunotherapy, oncological and hematological diseases, transplants, surgical procedures and acquired immunodeficiency syndrome (AIDS), among others (Singh et al., 2008; Henao-Martínez and Beckham, 2015; Liao et al., 2016). In addition, factors such as extremes of age and prolonged exposure to antimicrobial therapies are also associated with high rates of human fungal infections. An epidemiological study carried out in the United States revealed that in the period between 1979 and 2000, there was a striking 207% annual increase in sepsis caused by fungi (Martin et al., 2003).

Currently, emerging viruses like the pandemic SARS-CoV-2, which causes Coronavirus Disease 2019 (COVID-19), alert doctors to the possibility of increased co-infections associated with the physiological impairment of COVID-19 patients and its clinical management (Segrelles-Calvo et al., 2020c). The SARS-CoV-2 infection is characterized by an increase in pro-inflammatory cytokines and a decrease in anti-inflammatory cytokines, resulting in a state of cytokine storm syndrome. Recent studies have shown that the use of dexamethasone has reduced inflammation and the period of invasive mechanical ventilation and hospital mortality in severe COVID-19 patients (Sterne et al., 2020). However, the use of corticosteroids is a risk factor widely studied for the development of invasive mycoses in critically ill patients (Sinha et al., 2020). Although there is still not enough published statistical data, fungal co-infections in COVID-19 patients are present in a significant number of hospitalized individuals, leading to serious complications or even death. Co-infections have been described for *Aspergillus* spp. (Segrelles-Calvo et al., 2020b), *Trichosporon asahii* (Segrelles-Calvo et al., 2020a), *Candida* spp. (Al-Hatmi et al., 2020), and *Cryptococcus* spp. (Passerini et al., 2020).

Among the vast fungal species that cause systemic mycoses in humans, *Cryptococcus* spp., of which *Cryptococcus neoformans* and *Cryptococcus gattii* are the main representative of the genus, is capable of causing cryptococcosis, a disease with 223,100 clinical cases per year and approximately 181,000 deaths (Rajasingham et al., 2017; Williamson et al., 2017). The high rates of incidence and mortality caused by fungal infections, especially cryptococcosis, associated with the absence of an efficient therapy, have led to the search for novel diagnostic and therapeutic alternatives to control these diseases (Prado et al., 2009; Kronstad et al., 2012; Chen et al., 2014).

*Cryptococcus* spp. is a basidiomycete that presents itself as a haploid and spherical yeast surrounded by a polysaccharide (PS) capsule, a unique feature among eukaryotes (McFadden et al., 2006). Infection with *Cryptococcus* spp. is acquired through inhalation of dried spores or yeasts (Ellis and Pfeiffer, 1990). The lungs, therefore, acts as the primary site of infection (Barbosa et al., 2006). After the fungus enters the host's alveolar epithelium, the infection can take the latent form or manifest itself as the acute form of the disease (Goldman et al., 2010). The successful spread of the pathogen is due to the evasion capacity of the host's immune system, benefited by several mechanisms of adaptation, such as the production of a capsule, titan cells, melanin and urease, among others (Kronstad et al., 2012; Zaragoza, 2019).

*Cryptococcus* spp. manages to easily deceive the immune system in a human host with some degree of immunosuppression. After the establishment of pulmonary infection, the fungus reaches the lung parenchyma and subsequently the bloodstream, causing systemic infections with damage to various anatomical sites, such as skin, bones, eyes, prostate and/or genitourinary tract (Casadevall and Perfect, 1998). However, the main scenario of cryptococcosis is the colonization of the Central Nervous System (CNS) (Mitchell and Perfect, 1995; Mitchell et al., 1995), considered the most advanced and lethal manifestation of the disease.

The Willis polygon, a set of cerebral arteries that vascularizes the CNS, plays a crucial role in the spread of the fungus. The medium-sized cerebral arteries branch into smaller pial and arterioles arteries that run along the surface of the brain. The pial arteries are surrounded by smooth muscle, a layer of endothelial cells and an outer layer of leptomeningeal cells (Chang et al., 2004; Franco-Paredes et al., 2017). The Virchow-Robin space surrounds the walls of arteries, arterioles, veins, and venules as they flow from the subarachnoid space and, when penetrating the cerebral parenchyma, plays an important role in draining interstitial fluid (Zhang et al., 1990). *Cryptococcus* spp. colonizes the cerebrospinal fluid (CSF), in the perivascular spaces and in the brain parenchyma via transcellular crossing of the endothelial cells of the blood-brain barrier (BBB), but without affecting the integrity of the latter. Other strategies used by *Cryptococcus* spp. to enter the CNS is the use of mononuclear cells in the process known as “Trojan horse” where fungi pass through the BBB within infected phagocytes or cause the endothelial cell junctions in the BBB to rupture, allowing the passage of free fungi (Chen et al., 2003; Chang et al., 2004; Jong et al., 2008; Charlier et al., 2009; Casadevall, 2010; Dromer and Levitz, 2010; Vu et al., 2013; Santiago-Tirado et al., 2017; Zaragoza, 2019).

After reaching the CNS, *Cryptococcus* spp. can promote two distinct clinical manifestations and both are potentially fatal: (I) cryptococcal meningoencephalitis or parenchymal presentations in the context of advanced immunosuppression, the most common infection of the CNS, and (II) *cryptococcus*-related Immune Reconstitution Inflammatory Syndrome (IRIS), that occurs after the initiation of highly active antiretroviral therapies (Del Valle and Piña-Oviedo, 2006).

Cryptococcal IRIS is well-characterized in HIV-infected patients and is associated with significant rates of morbidity and mortality. In addition, Cryptococcal IRIS is estimated to occur between 5 and 11% in patients who received organ transplantation with cryptococcal infection and is associated with the increased risks of allograft failure (Morrison et al., 1994; Patterson, 1999). The clinical characteristics of cryptococcal IRIS are similar to the active cryptococcal infection itself, occurring more commonly as a CNS disease, with meningeal disease as the most serious presentation. Furthermore, lymphadenitis, pneumonitis, soft tissue involvement and, mediastinitis have also been reported. A differential diagnosis is the appearance of necrotic granulomatous inflammation with the presence of yeasts in histopathological analysis. Despite changes in inflammatory markers, there are no specific reliable tests for cryptococcal IRIS thus, establishing the diagnosis is a considerable clinical challenge, especially because of atypical presentations (Maziarz and Perfect, 2016).

Given the high frequency of glucocorticoid use in the clinic, when the patient has an inflammatory condition without a proper microbiological diagnosis, it becomes necessary to better evaluate the effects of this class of molecules during fungal infections. Glucocorticoids have shown short-term improvement in quality of life for patients with Cryptococcal IRIS due to their anti-inflammatory activity, thus apparently decreasing the need for hospitalization. However, they should not be used to prevent IRIS or to control intracranial pressure, since studies have associated their use with patients' death, although the causes of high mortality are not yet fully understood (Kuwahara et al., 2014; Beardsley et al., 2016). Some immunosuppressive characteristics induced by glucocorticoids are already known in cryptococcosis. For example, cortisone acetate has been shown to decrease the ability of alveolar macrophages to phagocytize *C. neoformans*, potentially leading to the spread of fungi in the bloodstream (Gross et al., 1996). Likewise, it substantially reduces the chemotactic activity of polymorphonuclear cells (PMNs) and monocytes in the cerebrospinal fluid, thus contributing to the subsequent inability to eradicate fungi with tropism to the CNS (Granger et al., 1985; Perfect and Durack, 1985a). Perfect and collaborators (Perfect et al., 1983) showed that glucocorticoids represent a high risk of cryptococcemia. Although some of the effects of these drugs on the host's immune system have been studied, little is known about the effect of corticosteroids on the metabolism and on the induction of virulence factors in *Cryptococcus* spp.

In the present work, we combined morphological and physicochemical characterization of *C. neoformans* capsular and its secreted PS together with histopathological analysis, survival curves, and interleukin modulation, to investigate the effects of dexamethasone (DX) or methylprednisolone (MP) on the metabolism and on the induction of virulence factors in *C. neoformans*. Our aim was to collect experimental evidence that provide a basis for future clinical administration of glucocorticoids in patients with cryptococcosis.

## Results

### Dexamethasone and Methylprednisolone Increase *C. neoformans* Proliferation and Surface Electronegativity but Reduce Capsule and Secreted PS Sizes

**Figure 1:**
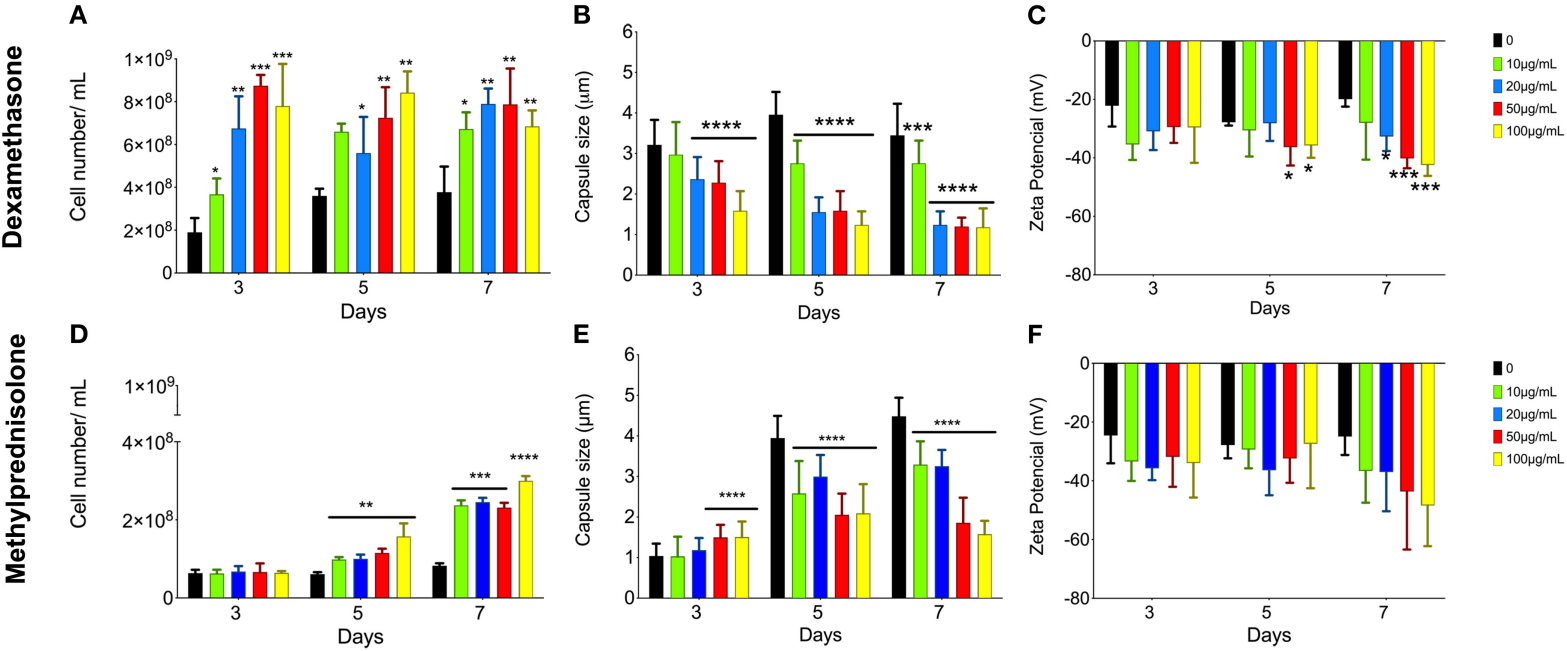
Effect of glucocorticoids on *C. neoformans* in growth, capsule size and Zeta potential (ξ). Yeast cells were grown in minimal media with 10 (green bars), 20 (blue bars), 50 (red bars), and 100 (yellow bars) μg/mL of dexamethasone **(A–C)** or methylprednisolone **(D–F)** for 3, 5, and 7 days. *p < 0.1; **p < 0.01; ***p < 0.001; ****p < 0.0001.

To analyze the effects of glucocorticoids on *C. neoformans*, yeast cells were allowed to grow in minimal media supplemented with different concentrations (10, 20, 50, and 100 μg/mL) of DX or MP for 3, 5, and 7 days (Figure 1). Cell proliferation, capsule size and electronegativity of the cell surface were evaluated.

The analyses of yeast cells grown in minimal media supplemented with different DX or MP concentrations showed an increase in *C. neoformans* proliferation when compared to control conditions, without the glucocorticoids (Figures 1A,D, respectively). This effect was more pronounced for DX. On day 3 of culture with DX, there was a significant increase in the number of cells for all tested concentrations (Figure 1A). For MP, the effect was slower, and the differences only began to be visualized after 5 days of treatment (Figure 1D).

Both DX and MP treatments induced a reduction in *C. neoformans* capsule size when compared to the controls, without glucocorticoids (Figures 1B,E). However, DX again showed stronger effects when compared to MP. A significant reduction in capsule size was observed at the day 3 with concentrations higher than 20 μg/mL of DX and became more pronounced as the length of treatment and DX concentration increased (Figure 1B). On the other hand, MP effects occurred more smoothly and upon 5 days of culture, where reductions in capsule size for all concentrations of glucocorticoids were observed (Figure 1E). It is worth mentioning that there was a slight increase in capsule size on day 3 and with 50 and 100 μg/mL of MP; however, this increase was reversed after day 5 onwards.

Changes in electronegativity of the cell surface were evaluated through Zeta potential analysis. Again, both DX and MP treatments showed increases in the surface electronegativity when compared to their respective controls (Figures 1C,F). For DX treatments, the Zeta potentials were more negative than the control from day 5 onwards and became more pronounced as the DX continued up to day 7 (Figure 1C). For MP treatments, the effects on Zeta potential slightly changed from the third day of cultivation; however, it became more pronounced on day 7 (Figure 1F).

**Figure 2:**
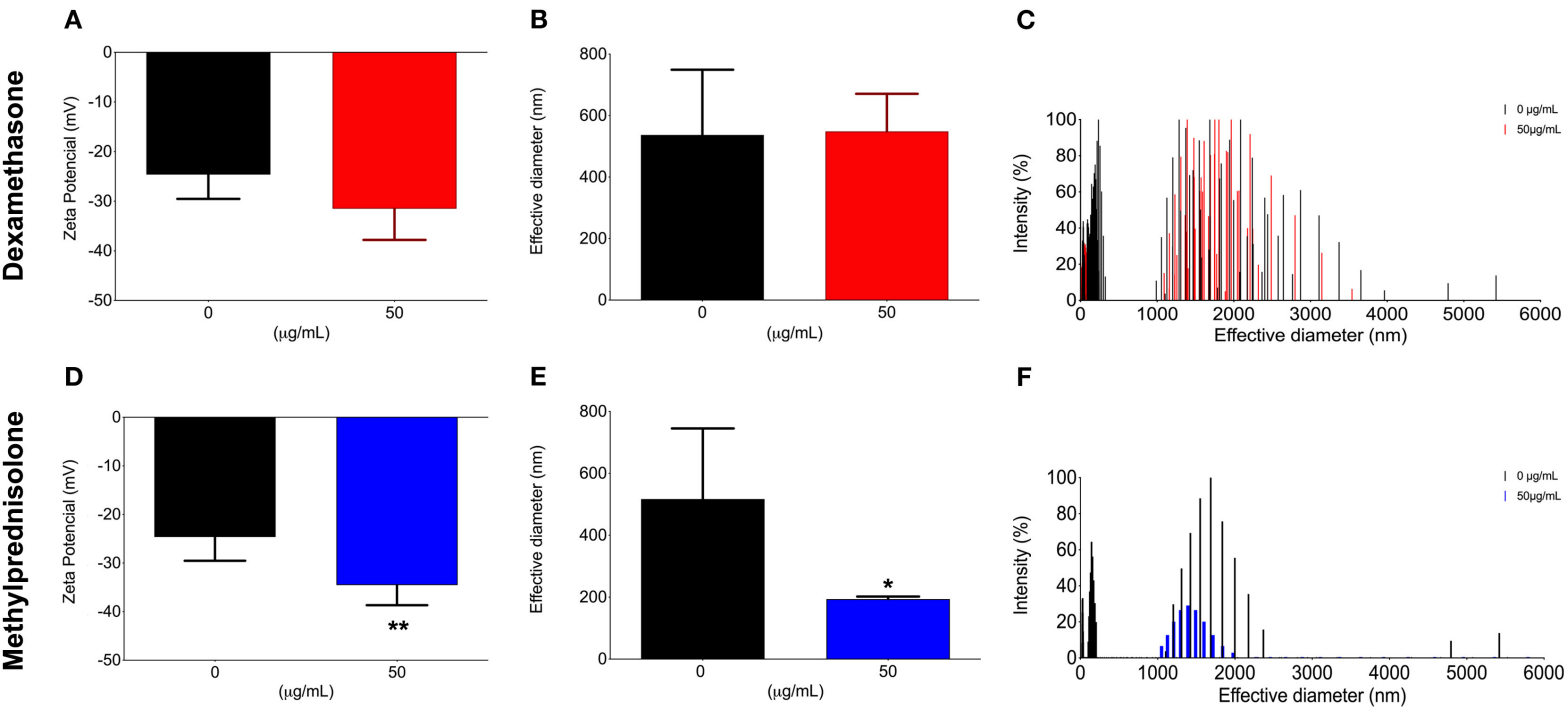
Effect of glucocorticoids on *C. neoformans* secreted polysaccharide l. Yeast cells were grown in medium minimum without corticoids (black bars, control) or 50 μg/mL of dexamethasone (**A–C**—red bars) or methylprednisolone (**D–F**—blue bars) for 3, 5, and, 7 days. *p < 0.1; **p < 0.01.

In order to evaluate the effect of both DX and MP glucocorticoids on *C. neoformans* secreted PS, we next decided to limit our observations on the concentrations of 50 μg/mL (Figure 2). We first measured the rate of PS production (total amount of secreted PS divided by the number of cells in culture). Control cells showed a production rate of 1.8 ng/cell. However, rates of 0.58 ng/cell and 1.1 ng/cell were obtained after treatments with 50 μg/mL of DX and MP, respectively. Surprisingly, the secreted PSs did not undergo significant changes in their electronegativities or in their sizes after treatment with DX (Figures 2A–C). In contrast, MP produced a significant change in the electronegativity of PS fibers (Figure 2D) and presented fibers with sizes 2.6-times smaller in terms of effective diameter than the secreted PS from control condition (Figures 2E,F).

### Dexamethasone and Methylprednisolone Decrease the Compactness of PS Fibers of *C. neoformans* Capsule

**Figure 3:**
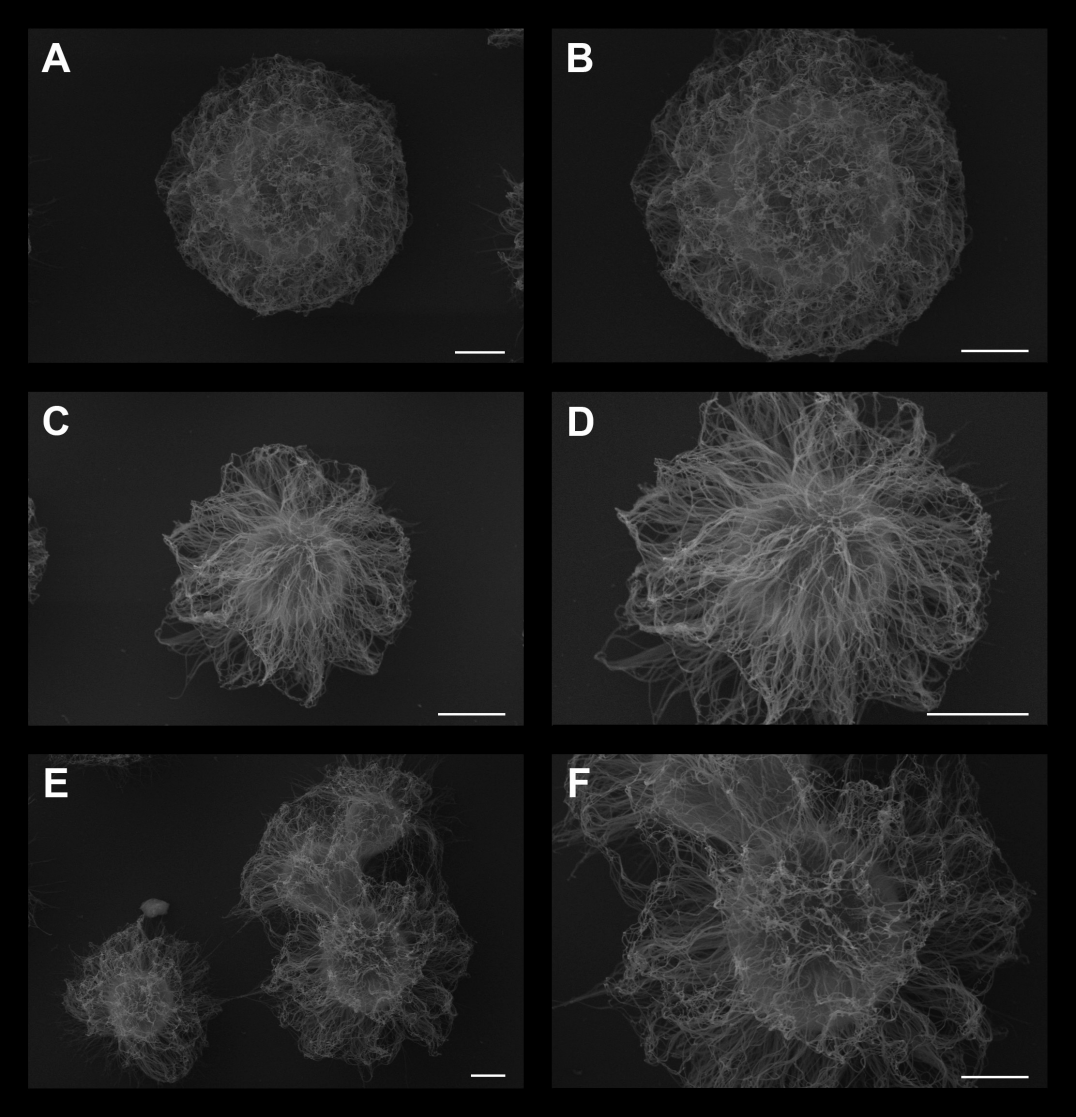
Scanning electron microscopy of *C. neoformans* grown in the presence of 50 μg/mL dexamethasone **(C,D)** or 50 μg/mL methylprednisolone (E and F panel). Control cells without treatment are presented in **(A,B)**. Scale bar: **(A,C,E)** 2 μm; **(B,D,F)** 1 μm.

Next, to observe possible changes in the ultrastructure of *C. neoformans* PS capsule, control cells or those treated with either DX or MP were processed and visualized by scanning electron microscopy (SEM) (Figure 3). Control cells presented more compact capsules, with a denser network of polysaccharides. Conversely, cells treated with DX (Figures 3C,D) or MP (Figures 3E,F) showed a loosened arrangement of the polysaccharide chains. This different feature allows the India ink to better penetrate in-between the empty spaces surrounding the PS fibers of both DX and MP conditions when compared to the control, thus explaining the apparent differences in capsule sizes shown in Figure 1.

### Dexamethasone and Methylprednisolone Increase the Survival Rate of *Galleria mellonella* Larvae Infected With *C. neoformans*

**Figure 4:**
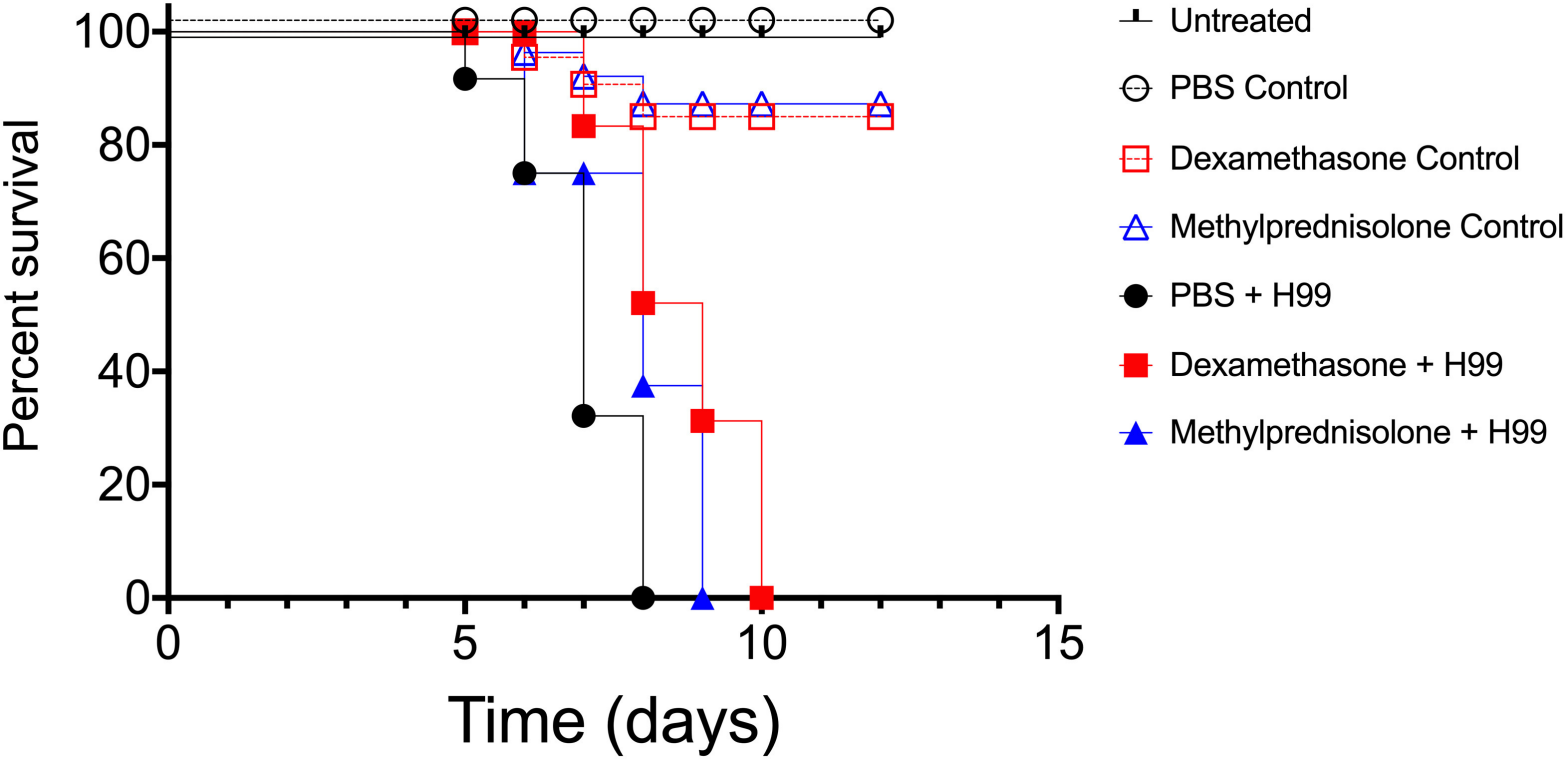
Infection model of *Galleria mellonella*. Survival curves of *G. mellonella* larvae infected with *C. neoformans* and treated with dexamethasone (DX–red square pictogram) our methylprednisolone (MP–blue triangle pictogram). Logrank and Gehan-Breslow-Wilcoxon tests *p* < 0.1 between filled black circles and filled blue triangles groups. *p* < 0.01 between filled black circles and filled red squares groups.

To assess possible influences on the virulence of *C. neoformans* after treatments with either DX or MP, we utilized the invertebrate host *G. mellonella*. This model has been used to study not only *C. neoformans* virulence but also the action of antifungals against this pathogen (Araujo et al., 2012; Araújo et al., 2017). All infected and untreated larvae died by day 8 upon infection (Figure 4). However, infected larvae previously injected with either DX or MP showed an overall prolongation on survival, with all subjects dying at day 10 and day 9, respectively, for DX (Square pictograms in red) and MP (Blue triangles pictograms), with significant differences in survival rates between treated groups and their respective controls.

### Effects of Dexamethasone and Methylprednisolone on the Survival, Spread, and on Changes in the Pattern of Cytokines in Mice Infected With *C. neoformans*

**Figure 5:**
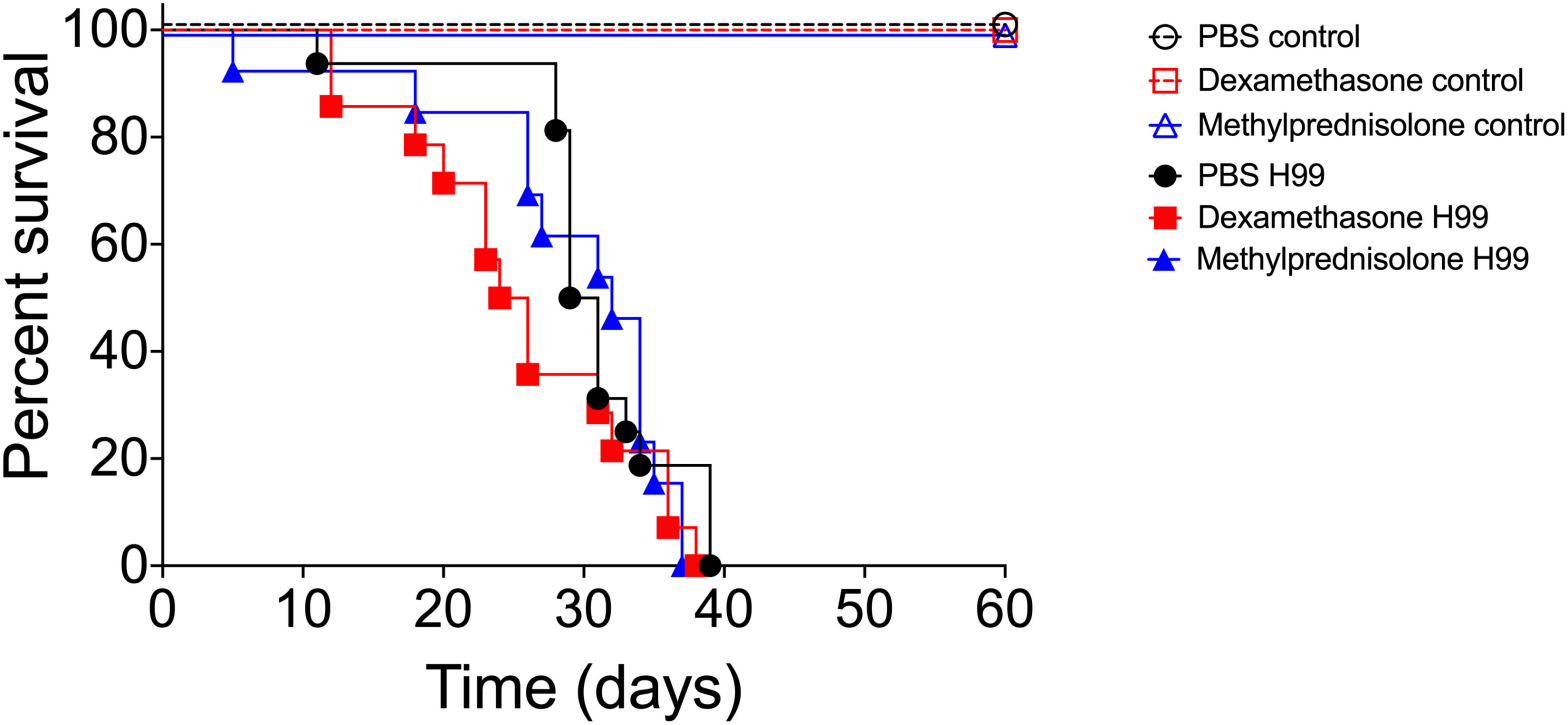
Infection model of Balb/c mice. Pictograms: black opened circle, red opened square, and blue opened triangle lines are controls. Black closed circle lines represented mice infected with *C. neoformans*; red closed square lines are mice infected with *C. neoformans* and treated with DX; blue closed triangle lines are mice infected with *C. neoformans* and treated with MP.

Given the high frequency of glucocorticoid use in the clinic, when the patient has an inflammatory condition without a proper microbiological diagnosis, we next decided to investigate the pattern of *C. neoformans* infection in mice by following infected animals undergoing treatment with either DX or MP without any administration of associated antifungals. First, we tested whether DX or MP treatments could provide protection against infection by *C. neoformans*. Balb/C mice were treated with either DX or MP to later be infected with *C. neoformans* or treated with PBS, as a control. We observed that control mice (PBS, MP, and DX) maintained 100% survival after day 60 (Figure 5). Infection with *C. neoformans* (without DX or MP) led to the death of all animals on day 39 after infection (Figure 5). For infected mice that were treated with either DX or MP, the death of all animals occurred on days 38 and 37, respectively (Figure 5). Although statistical analysis did not show significant differences between the groups, infected animals treated with either DX or MP started to die earlier than the untreated animals, especially DX, indicating a possible change in the modulation of the infection.

**Figure 6:**
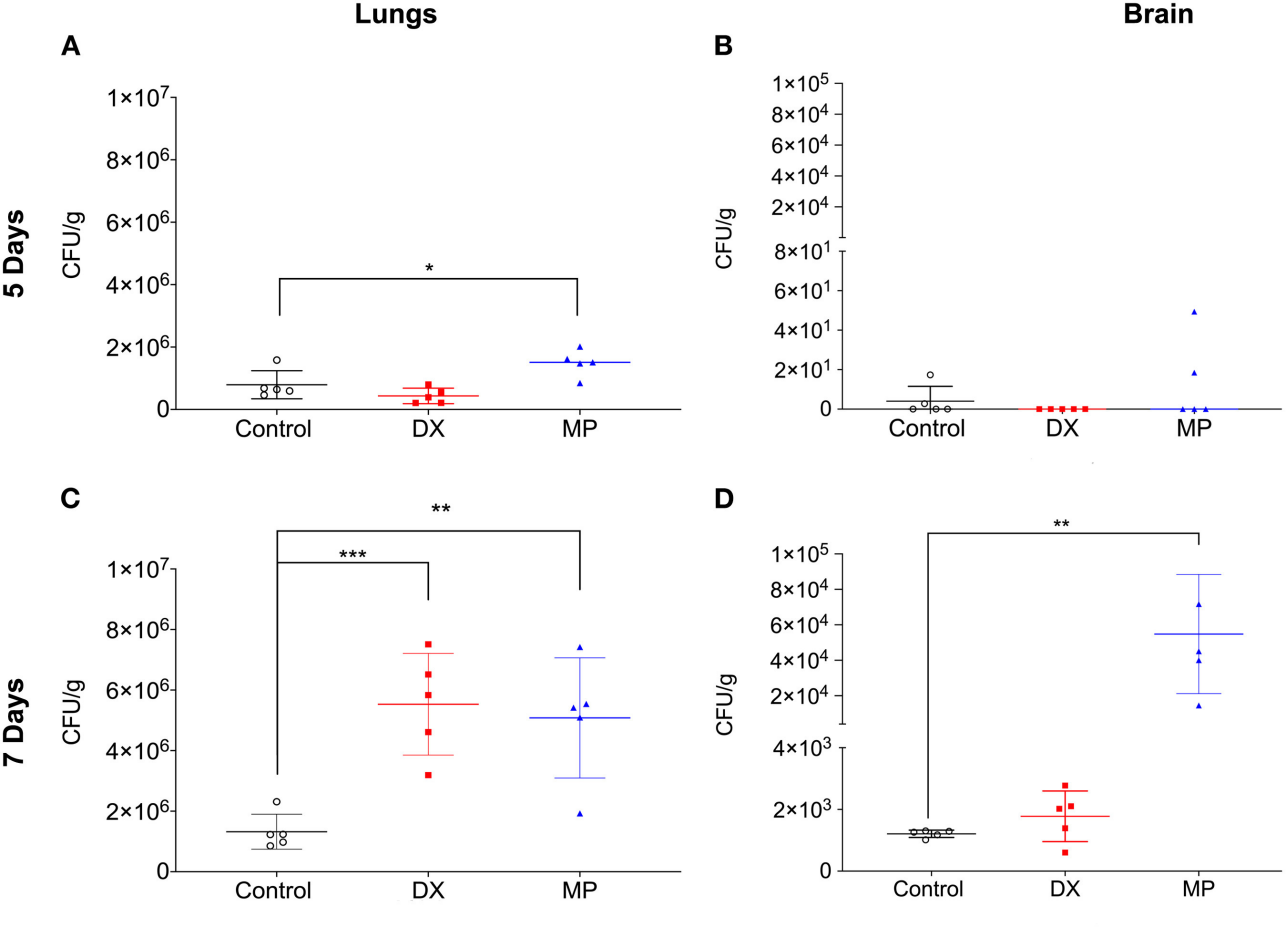
Colony-forming units (CFU) in the lungs **(A,C)** and brain **(B,D)** of Balb/c mice. DX represented mice infected with *C. neoformans* and treated with dexamethasone (DX; MP are mice infected with *C. neoformans* and treated with methylprednisolone (MP). CFU were analyzed after five **(A,B)** and seven **(C,D)** days of infection. *p < 0.1; **p < 0.01; ***p < 0.001.

Subsequently, we tested whether the *in vivo* administration of either DX or MP could alter the pathogenesis of the spread of cryptococcosis. In our model, the fungus spread was evaluated after 5 and 7 days of infection. On day 5, mice that received the DX treatment showed similar numbers of lung colony forming units (CFUs) as those from the control (Figure 6A, 4.3 ± 2.5 × 10^5^ vs. 7.9 ± 4.4 × 10^5^ CFUxg^−1^, respectively). A similar behavior was observed for cerebral CFUs and its control (Figure 6B, 0.0 ± 0.0; 4.0 ± 7.5 CFUxg^−1^, respectively). However, infected mice that received the MP treatment presented lung CFUs significantly different from its control (Figure 6A, 1.4±0.4 × 10^6^ vs. 7.9 ± 4.4 × 10^5^ CFUxg^−1^, respectively—*p*= 0.0342) but no difference was observed between the cerebral CFU of MP treatment and its control (Figure 6B, 13.6 ± 21.5; 4.0 ± 7.5 CFUxg^−1^, respectively).

On day 7 after infection, the mice that received the DX treatment showed lung CFUs significantly larger than its control (Figure 6C, 5.5 ± 1.6 × 10^6^ vs. 1.3 ± 0.5 × 10^6^ CFU/g^−1^, respectively) but no significant difference between cerebral CFUs and its control (Figure 6D, 1.2 ± 0.8 × 10^3^ vs. 1.2 ± 0.1 × 10^3^ CFUxg^−1^, respectively). In turn, mice that received MP treatment demonstrated lung CFUs significantly higher than control (Figure 6C, 5.1±1.9 × 10^6^ vs. 1.3 ± 0.5 × 10^6^ CFUxg^−1^, respectively). MP treatment also significantly increased cerebral CFUs compared to its control, (Figure 6D, 5.4 ± 3.3 × 10^4^ vs.1.2 ± 0.1 × 10^3^ CFUxg^−1^, respectively).

**Figure 7:**
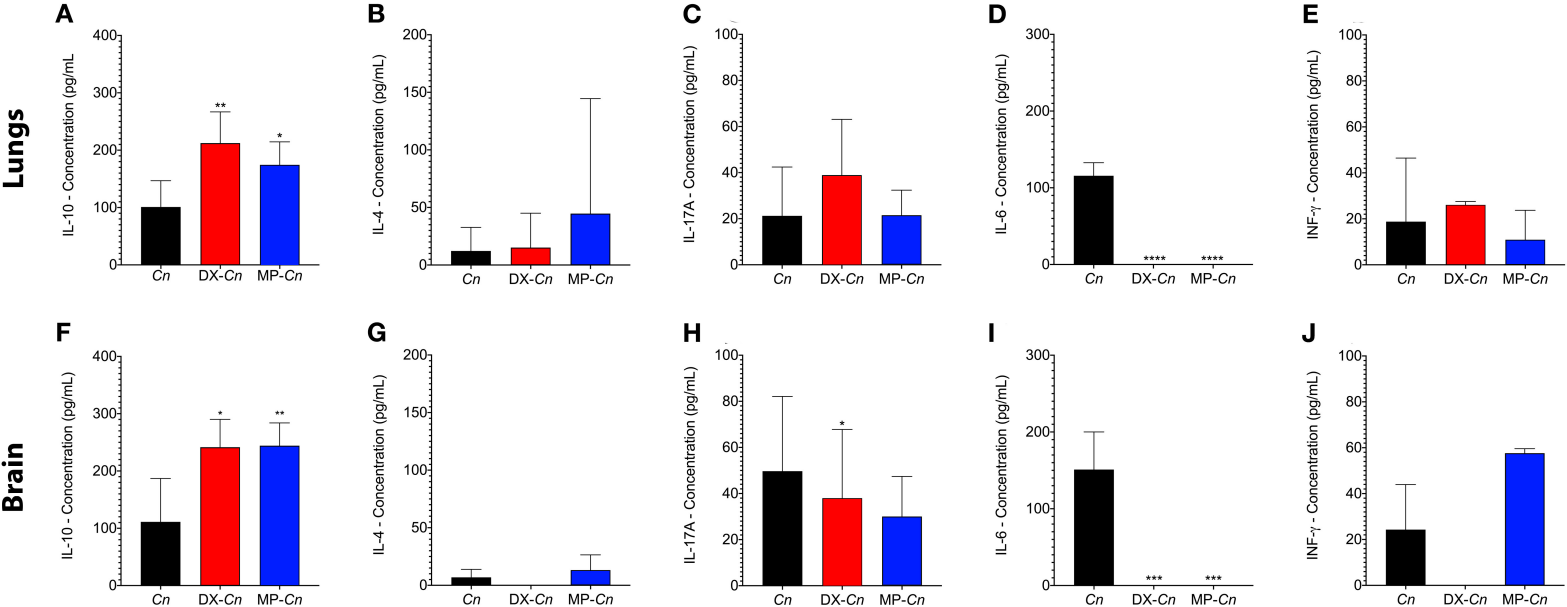
Comparisons of expressions of IL-4, IL-10, IL-17A, IL6, and INF- γ in lungs and brain of animals treated with dexamethasone (DX) and methylprednisolone (MP) at 7-days post infection. Cn: *C. neoformans* control; DX-CN: *C. neoformans* treated with DX; MP-Cn: *C. neoformans* treated with MP. *p < 0.1, **p < 0.01, ***p < 0.001, and ****p < 0.0001.

To investigate the secretion pattern of different immune system modulators (IL-10, IL-4, INF-γ; IL-6 and IL-17), the lungs and brain homogenates of either control or infected mice were analyzed after 7 days of infection (Figure 7). The mice that received the selected doses of DX or MP showed a statistically significant increase in pulmonary and cerebral IL-10 production when compared to the controls (Figure 7), whereas the IL-4 and IL-17 levels in either lungs and brains did not show significant differences among groups. Moreover, when the anti-inflammatory IL-6 was followed, we observed that both DX and MP treatments induced a drastic reduction in its pulmonary and cerebral levels (Figure 7). For the pro-inflammatory INF-γ however, no significant differences were noticed among controls, DX or MP treatments in lungs and brains (Figure 7).

**Figure 8:**
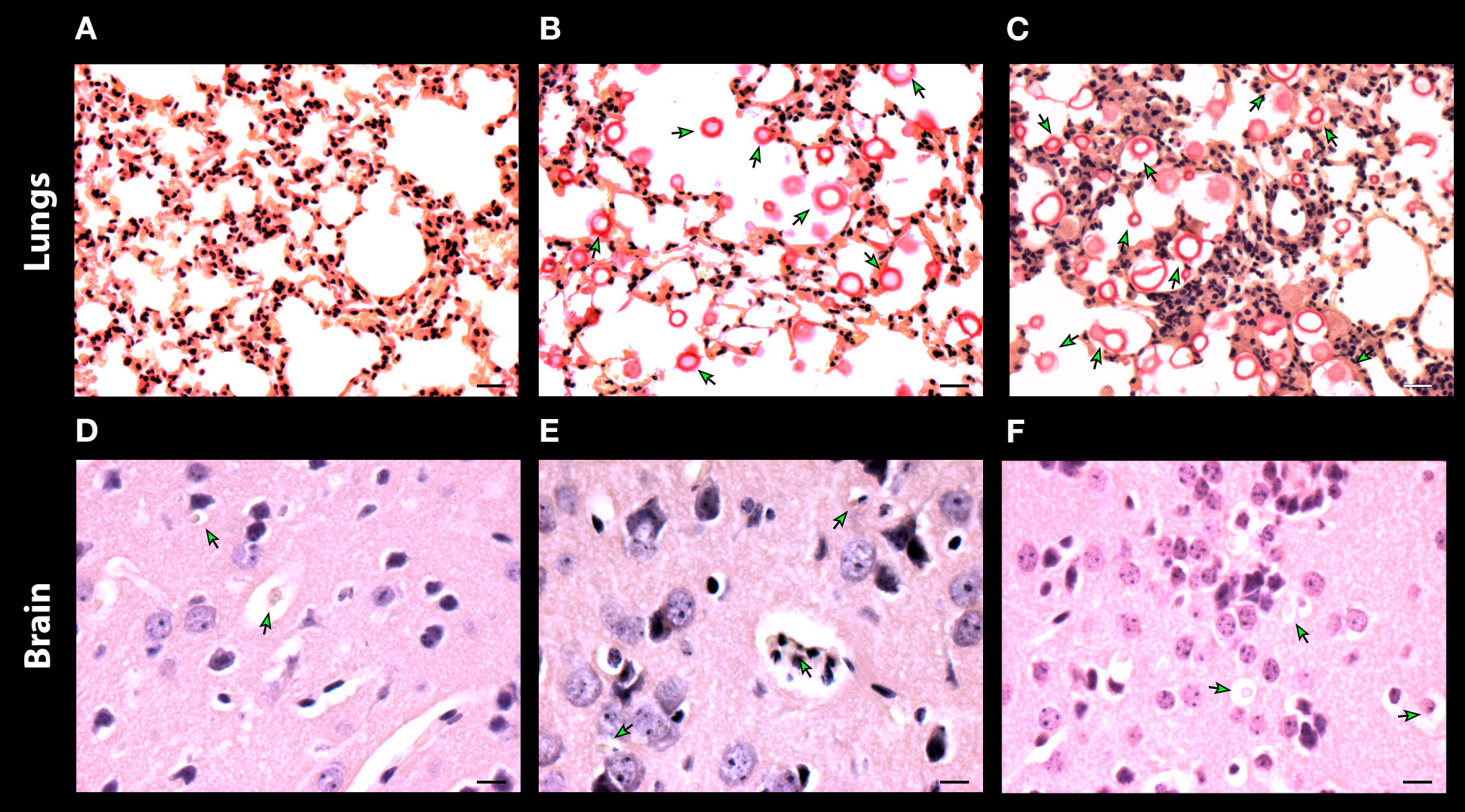
Histopathology of lungs **(A–C)** and, brain **(D–F)** of mice infected with *C. neoformans*. Control without treatment **(A,D)** and treated with dexamethasone **(B,E)** and methylprednisolone **(C,F)**. Scale bar 10 μm.

To corroborate and also to better visualize the inflammatory changes and the presence of fungal cells in the lung and brain after 7 days of infection, samples of these organs were preserved and stained with mucicarmine (Figure 8). The presence of inflammatory cells was visualized in lung tissues of mice infected with *C. neoformans* cells, with presence of few yeasts (Figure 8A). Contrarily, the mice treated with either DX or MP showed a reduction of inflammatory cells but a strong increase in the number of encapsulated fungal cells (Figures 8B,C, respectively). For brain tissues, both the control group and those mice treated with DX presented a great number of inflammatory cells and few yeast elements (Figures 8D,E). However, the mice treated with MP showed a fair reduction in the number of inflammatory cells and an intense increase in number of fungal cells (Figure 8F).

## Discussion

Cryptococcosis is an important systemic fungal disease, threatening the lives of humans and other animals, predominantly due to pulmonary and CNS alterations. It usually affects immunocompromised individuals, mainly those with HIV, presenting itself as an opportunistic infection, although other predisposing conditions have been described, including treatment with immunosuppressants, organ transplants, lymphoproliferative disorders and, neoplasms (Mitchell and Perfect, 1995; Perfect and Casadevall, 2002). Estimates show that almost 45% of seropositive individuals in advanced immunosuppression stages succumb as a result of cryptococcosis (Clumeck et al., 1984; Van De Perre et al., 1984; Park et al., 2009; Rajasingham et al., 2017). In a recent demographic study among the population of South Africa, individuals who received antiretroviral treatment had rates of Cryptococcus infection of 95 cases/100,000 inhabitants while individuals with advanced immunosuppression had about a 10 times higher prevalence (McCarthy et al., 2006).

The main manifestations of cryptococcosis include respiratory infections, since the lungs are the agent's first entrance into the host, through the inhalation of propagules present in the environment in the form of dehydrated yeasts, while the CNS is the destination due to the fungus neurotropism (Galanis et al., 2010). In lungs, cryptococcosis varies from a simple colonization of the airways in asymptomatic patients to an acute respiratory distress syndrome with severe respiratory failure, in patients with deficiency in cellular immunity (Henson and Ross Hill, 1984; Vilchez et al., 2001). In the CNS, cryptococcosis shows well-known manifestations of meningitis and meningoencephalitis, and its evolution is generally subacute or chronic (Graybill et al., 2000). Although some HIV patients present minor symptoms due to a high degree of immunosuppression; this does not reduce the high morbidity and mortality of the disease (Perfect and Casadevall, 2002).

Moreover, when a patient has an inflammatory condition, without a known infection, the administration of glucocorticoids is quite common. However, the effects of these molecules during cryptococcosis have been investigated. For example, cortisone acetate has been shown to decrease the ability of alveolar macrophages to bind and ingest *C. neoformans*, potentially leading to the spread of fungi in the bloodstream (Gross et al., 1996). Previous clinical work has shown that glucocorticoids represent a high risk of cryptococcemia. In addition, glucocorticoids predispose patients with cancer or sarcoidosis and recipients of allogeneic bone marrow and solid organ transplants to cryptococcosis. The administration of glucocorticoids reduces the chemotactic activity of cerebrospinal fluid in relation to polymorphonuclear leukocytes (PMNs) and monocytes (Perfect and Durack, 1985b). This factor may contribute to the significant lack of influx of PMN into the cerebrospinal fluid and subsequent inability to eradicate fungi with tropism in the CNS, such as *C neoformans*. Such attenuation is further intensified by abnormalities induced by glucocorticoids in microglial cells. Glucocorticoids are also a critical factor for the outcome of this infection. Diamond and Bennet reported that recurrences of cryptococcal meningitis were associated with the maintenance of glucocorticoid treatment, in addition to the termination of antifungal therapy (Diamond, 1974).

Although some effects of glucocorticoids on the clinic are already known, their effects on the metabolism and induction of virulence factors in *Cryptococcus neoformans* were less studied. Herein, we observed how *C. neoformans* cells behave after being in contact with either DX or MP, particularly focusing on the biological aspects of this fungus. We demonstrated that all the used concentrations of either DX and MP induced an increase in cell proliferation of *C. neoformans*, but the effect was more prominent for DX than for MP. In addition, either DX and MP treatments induced an apparent reduction in *C. neoformans* capsule size when compared to the control. Deeper observations using scanning electron microscopy showed that these reductions were probably correlated to modifications in PS fiber morphology, that changed from denser and more compacted capsules in control condition, to capsules with more sparse PS fibers under DX and MP treatments, therefore possibly allowing greater penetration of India ink, which gave the apparent impression of smaller capsule size. Indeed, differences in capsule ultrastructural morphology were recently described using scanning electron microscopy (Lopes et al., 2020). Although the study from Lopes et al. showed that several capsule morphologies are present in the same population, we conjecture that a global change in phenotype, tending to one of the morphologies could occur after a population is confronted with a specific external agent, for example, the glucocorticoids used in our present study. However, it remains to be investigated how these possible variations affect *C. neoformans* virulence as well as the clinical symptoms.

We also observed a reduction in *C. neoformans* PS production, from 1.8 ng/cell in control to 0.58 ng/cell after DX treatment. Surprisingly, these secreted PSs did not undergo significant changes in electronegativity and the polysaccharide fibers dimension remained unaltered. In contrast, the MP treatment did not induce a significant change in PS production but showed significant alterations in the electronegativity of secreted PS, with more negative Zeta potentials compared to control cells, and size of fragments, with values 2.6-times smaller in terms of effective diameter. Previous results from our group have demonstrated that the biological properties of PSs are greatly influenced by their size and physicochemical properties, implying that structural parameters can not only alter their biological functions but can also induce different host responses (Frases et al., 2009a,b; Albuquerque et al., 2014; Pontes and Frases, 2015; Araújo et al., 2016, 2017, 2019). Overall, our results suggest that changes in the size, morphology, and electronegativity of the capsule and secreted PSs can affect the fungal pathogenesis, producing different dissemination patterns due to the influence of corticosteroids in these structures. Our results also argue in favor of the general assumption that corticosteroids, while helping during the inflammation process, can also compromise the patient's prognosis by increasing the fungal load and reshaping the PS structure to possibly more active forms.

Moreover, it is already known that glucocorticoids decrease the number of macrophages, their phagocytic capacity and antigen processing as well as their recruitment to sites of inflammation (Balow and Rosenthal, 1973; Keil et al., 1995; Xie et al., 2019). In addition to the effects that these drugs have on the host's phagocytic cells, we are showing in the present study that DX and MP also influence the secreted capsule and polysaccharide sizes and electronegativity. Changes in capsule size, flexibility and electronegativity have been described as capable of influencing the capacity of macrophages to phagocytize *C. neoformans* (Frases et al., 2011; Albuquerque et al., 2014; Pontes and Frases, 2015; Ding et al., 2016; Araújo et al., 2017). Thus, we conjecture that the effects of DX and MP would probably reduce the ability of macrophages to phagocytize *C. neoformans*, not only due to the direct effects of these drugs on macrophages (Balow and Rosenthal, 1973; Keil et al., 1995; Xie et al., 2019), but also because the alterations in the fungus surface would probably lead to a decrease in phagocytosis if compared to the changes already reported in the literature (Frases et al., 2011; Albuquerque et al., 2014; Pontes and Frases, 2015; Ding et al., 2016; Araújo et al., 2017). Nevertheless, future studies would be essential to confirm this hypothesis.

Our results also warn that the “indiscriminate” use of corticosteroids, without consolidating the correct antifungal treatment, can lead to the death of patients in a similar or even faster than in their absences, since we observed no significant differences with regards to the overall survival between both untreated and treated mice groups, but also a trend of increasing mortality in the first days. We conjecture that the 5-day corticosteroid treatment window concomitant with *C. neoformans* infection, was probably responsible for causing the initial death acceleration in mice observed at the beginning of the survival curves (Dexamethasone H99 and Methylprednisolone H99), when compared to that of control (PBS H99). However, as the corticosteroid treatment was interrupted in the following days, the survival curves show a tendency to approach that of control. Altogether, the survival curves in mice indicate a possible change in the modulation of infection, probably due to the increase in fungal proliferation in the first days caused by the corticosteroid treatment, and this increase in proliferation allows a faster dissemination through the hematological route (Wilson et al., 1970).

In contrast, the *G. mellonella* model shows an opposite behavior. *G. mellonella* larvae have been used extensively to investigate virulence properties of fungi and the relevance of the innate immune response during fungal infections (Mylonakis et al., 2005; Jemel et al., 2020; Wojda et al., 2020). Although this result could be considered unexpected, this is not the first report showing that *C. neoformans* virulence attributes have a distinct profile when comparing mice and insect models. Eisenman and colleagues (Eisenman et al., 2014) have shown that larvae of *G. mellonella* infected with melanized yeasts of *C. neoformans* lived longer than that infected with non-melanized fungi. Thus, it is possible that the modified capsule and GXM could promote a higher activation of innate immune cells, increasing the insect resistance. Further studies are required to confirm this hypothesis.

We also analyzed whether DX or MP could alter the pathogenesis of the spread of cryptococcosis. We observed that in the acute phase, mice that received treatment with DX presented a similar fungal load to control in lungs and brain. For MP, mice that received the treatment had pulmonary CFUs significantly higher than controls but showed no differences in their CNS. However, the described scenario completely changed during the chronic phase. The mice that received treatment with DX started to present pulmonary CFUs significantly higher than their controls, whereas cerebral CFUs displayed similar levels. For MP, the mice that received treatment had pulmonary and cerebral CFUs significantly greater than their respective controls.

Finally, we also analyzed the pattern of cytokine expression for mice who received treatments with either DX or MP. A statistically significant increase in the production of IL-10 was observed for both treatments, when compared to the control, indicating a possible Th2 response, despite the unchanged levels of anti-inflammatory IL-4. In turn, the production of IL-17 did not show significant differences between the treated groups (DX or MP) and organs (lungs and brain). A striking observation was that animals treated with DX or MP prior to infection suffered a drastic reduction in the lungs and the brain levels of IL-6 when compared to the control, despite its role as pro-inflammatory cytokine and antagonist to regulatory T cells and regulating the production of IL-1 and TNF-α. In the case of INF-γ, no significant differences were observed between the control mice and those treated with DX and MP. The immunosuppressive response to the increase in IL-10, together with a reduction in IL-6 levels, supports the spread of *C. neoformans* infection. IL-6 is a cytokine that influences the blood-brain barrier integrity by reducing the blood-brain barrier permeability during *Cryptococcus* meningitis (Li et al., 2015). Our results show a decrease in the presence of IL-6 in the treated mice, which may provide an explanation for the higher values of dissemination in these groups.

## Conclusions

Several studies have demonstrated the role of glucocorticoids in microbial infection focusing on their role in the host. However, the present study demonstrated that changes in the ultrastructure and polysaccharide architecture, influenced by glucocorticoids, may further support the spread and severity of the infection.

Nowadays the use of glucocorticoids, such as hydrocortisone, dexamethasone and methylprednisolone, in the treatment of inflammatory diseases such as COVID-19, has reduced mortality by almost one third among patients who need respirators and in about one fifth among patients who require only oxygen therapy. However, the number of fungal infections associated with COVID-19 is increasing. Our results bring a medical alert to the indiscriminate use of glucocorticoids that may trigger physiological impairment in patients at risk for cryptococcosis.

## Methods

### *Cryptococcus* Strain

*C. neoformans var. grubii* H99 (ATCC 208821, clinical isolate), donated by Prof. Arturo Casadevall (Johns Hopkins Bloomberg School of Public Health, Baltimore, Maryland, USA) was used for all the experiments of this work. Yeasts were maintained in glycerol stocks at −80°C and grown on Sabouraud media at 30°C.

### Glucocorticosteroids and Growth Conditions

Yeasts were grown in minimal medium (15 mM glucose, 10 mM MgSO_4_7H_2_O, 29 mM KH_2_PO_4_, 13 mM glycine, and 3 μM thiamine, pH 5.5) supplemented with 10, 20, 50, and 100 μg/mL of methylprednisolone sodium succinate (CAS Number:83-43-2, Sigma Aldrich) or dexamethasone acetate (CAS Number: 50-02-2, Sigma Aldrich), at 37°C, for 3, 5, and 7 days. Cells grown in minimal media without drugs were used as controls. After growth, the cells were obtained by centrifugation (6,708 × *g* for 5 min), with subsequent determination of growth rates performed by CFUs counting in Sabouraud media.

### Capsule Size

To measure capsule thickness, cells from all experimental conditions used in this study were centrifuged at 6708 x *g* for 5 min, negatively stained with India ink and then imaged in an AXIO Lab.A1 light microscope (ZEISS, Germany). The capsule thickness (i.e., the distance between the cell wall and the outer limit of the capsule) was measured from a minimum of 100 cells, using the ImageJ software 1.8.0g (http://rsb.info.nih.gov/ij/. National Institutes of Health (NIH), Bethesda, MD).

### Scanning Electron Microscopy (SEM)

In brief, *C. neoformans* cells grown for 7 days at 50 μg/mL were washed three times in PBS pH 7.4 and fixed in 2.5% glutaraldehyde solution grade I (Electron Microscopy Sciences, Hatfield, PA, USA) in sodium cacodylate buffer 0.1 M pH 7.2 for 1 h at room temperature. Then, the cells were washed three times in 0.1 M sodium cacodylate buffer pH 7.2 containing 0.2 M sucrose and 2 mM MgCl_2_ (Merck Millipore Darmstadt, Germany), and adhered to 12 mm diameter round glass coverslips (Paul Marienfeld GmbH Co. KG, Germany) previously coated with 0.01% poly-L-lysine (Sigma-Aldrich, Darmstadt, Germany) for 20 min. Adhered cells were then gradually dehydrated in an ethanol (Merck Millipore, Darmstadt, Germany) series (30, 50, and, 70% for 5 min and 95 and 100% twice for 10 min). The coverslips were then critical-point-dried using an EM DPC 300 critical point drier (Leica, Germany) and mounted on specimen stubs using a conductive carbon adhesive (Pelco Tabs™, Stansted, Essex, UK). Next, the samples were coated with a thin layer of gold-palladium (10-15 nm) using the sputter method (Balzers Union FL−9496, Balzers, FL) (Carl Zeiss Evo LS or FEI Quanta 250), operating at 10–20 kV.

### Purification of Secreted Polysaccharides

Secreted capsule polysaccharides (secreted PS) from cells grown for 7 days at 50 μg/mL of MP or DX were purified by ultrafiltration using an Amicon^®^ (Merck KGaA, Darmstadt, Germany) system with a cutoff of 10 kDa (Millipore, Danvers, MA), as described previously (Nimrichter et al., 2007). The concentration of polysaccharides in filtered solutions was determined by the phenol-sulfuric method (Dubois et al., 1951). Glucose solutions were used as a standard. Cells grown for 7 days in the absence of glucocorticoids were used as controls.

### Calculation of the Effective Diameter and Hydrodynamic Radius of the Secreted PS Samples by Dynamic Light Scattering (DLS)

The effective diameter and polydispersity of the PS from cells grown for 7 days at 50 μg/mL of MP or DX were determined by dynamic light scattering on a NanoBrook Omni particle equipment (Brookhaven Instruments Corporation, Holtsville, NY) from a 1 mg/mL solution (Araujo et al., 2012; Araújo et al., 2016, 2017, 2019). Cells grown for 7 days in the absence of glucocorticoids were used as controls.

### Zeta Potential Measures (ξ)

The Zeta potential of PS samples at 1 mg/mL, from cells grown for 7 days at 50 μg/mL of MP or DX, were calculated on a Zeta potential analyzer (NanoBrook Omni particle, Brookhaven Instruments Corporation, Holtsville, NY) (Araujo et al., 2012; Araújo et al., 2016, 2017, 2019).

### *Galleria mellonella* Infections

*G. mellonella* larvae were selected according to size, mass, and the absence of any pigmentation marks for reproducible results. First, infection area was cleaned with 70% ethanol using a cotton swab. Larvae with initial mass of 300 ± 3 mg was previously treated with MP (30 mg/kg), DX (2 mg/kg) or PBS control for 2 days before infection. Subsequently, animals were inoculated with 10 μL of a yeast suspension prepared with 10^5^ cells (10^7^ cells/mL) through an injection in the last left proleg using a 26G gauge needle with Hamilton syringes. Ensuing to infection, larvae continued to receive daily doses of DX, MP or PBS for 5 days. In order to mitigate possible damage caused by successive injections, pharmacological administration was alternated between the last prolegs (right and left), the same occurred with non-pharmacological groups.

After the injection, the caterpillars were placed in 90 mm glass plates and incubated at 37°C. The number of dead caterpillars was monitored daily. The groups evaluated were: **(I)** Sham (without any treatment or manipulation); **(II)** PBS—Negative control (manipulative effect); **(III)** DX control (pharmacological control group without infection); (IV) MP control (pharmacological control group without infection); **(V)** Treated with DX and subsequently infected with *C. neoformans*; **(VI)** Treated with MP and subsequently infected with *C. neoformans* and **(VII)** Infected with *C. neoformans* alone (infection control without treatment with glucocorticoid). Survival curves were plotted using GraphPad Prism 9.0.0 (La Jolla, CA, USA). Each experiment was repeated at least twice.

### Mice Infections

Female Balb/c mice (*Mus musculus*) aged 6–8 weeks, with a mass of 20–25 grams, were previously treated with MP [30 mg/kg, intraperitoneally (IP)], DX (2 mg/Kg, IP) or PBS control for 2 days prior to infection. The infection with *C. neoformans* occurred at the third day upon treatment with the respective glucocorticoids. *C. neoformans* were injected intratracheally with 50 μL of an initial inoculum at 10^6^ cells/mL. Further treatment for an additional 5 days with MP (30 mg/kg, IP) or DX (2 mg/kg, IP) were administrated to evaluate the survival rate. In parallel, mice were also evaluated for fungal load, cytokine profile, and histopathology of the main organs (lungs and brain) affected during the infection.

The groups evaluated were: **(I)** Sham (without any treatment or manipulation); **(II)** PBS–Negative control; **(III)** DX control (pharmacological control group without infection); **(IV)** MP control (pharmacological control group without infection); **(V)** Treated with DX and subsequently infected with *C. neoformans*; **(VI)** Treated with MP and subsequent infected with *C. neoformans* and **(VII)** infected with *C. neoformans* alone (infection control without glucocorticoid treatment). All animals were maintained under optimal conditions. For survival tests, the death of animals was assessed daily, as well as the signs and symptoms of the disease. For the dissemination tests and impact of treatment on organ fungal load, after 5 and 7 days of infection, the animals were euthanized, and the lungs and brain were removed. A small fragment was separated for histopathological analysis upon fixation and staining with hematoxylin-eosin and mucicarmine stain. The remaining organ was homogenized and plated on Sabouraud media plates for CFU enumeration. For the analysis of selected cytokines, organs were centrifuged at 5,000 *g*, the supernatants from lungs and brains were collected and the presence of cytokines determined using the “RayBio^®^ Mouse Cytokine Antibody Array” kit (RayBiotech, Inc), following the manufacturer's instructions. Positive reactions were quantified using the Scion Image software (2000 Scion Corporation, NIH).

### Statistical Analysis

All the data, except those from the survival curves, are presented as mean ± standard deviation. Data were analyzed using GraphPad Prism 9.0.0 (GraphPad Software, San Diego, California USA, www.graphpad.com). Student's *t-*test were used for comparisons between each situation and the respective control. Survival curves were analyzed using the Logrank and Gehan-Breslow-Wilcoxon tests. *p < *0.05*; **p < *0.01*; ***p < *0.001*, and ****p < *0.0001*. The *p-values* and other numbers for all experiments are provided in the figure legends.

## Data Availability Statement

The raw data supporting the conclusions of this article will be made available by the authors, without undue reservation.

## Ethics Statement

The animal study was reviewed and approved by the Ethics Committee on the Use of Animals in Scientific Experimentation (CEUA) at the Health Sciences Center of the Federal University of Rio de Janeiro, registered with the National Council for the Control of Animal Experimentation (CONCEA) under the protocol number 01200.001568/2013-87 (112/17).

## Author Contributions

GA: conceptualization, methodology, investigation, visualization, data curation, formal analysis, writing—original draft, and writing—review & editing. VA, PM-d-S, HL, and CT: methodology and writing—review & editing. AG and LN: conceptualization, methodology, investigation, visualization, data curation, resources, supervision, formal analysis, writing—original draft, and writing—review & editing. BP and SF: conceptualization, methodology, investigation, visualization, data curation, resources, supervision, funding acquisition, formal analysis, writing—original draft, and writing—review & editing. All authors contributed to the article and approved the submitted version.

## Conflict of Interest

The authors declare that the research was conducted in the absence of any commercial or financial relationships that could be construed as a potential conflict of interest.
